# Quality and stability evaluation of Guizhou spicy chicken treated with gamma irradiation during the storage period

**DOI:** 10.1002/fsn3.3232

**Published:** 2023-01-18

**Authors:** Guolin Li, Ping Lin, Yongfu Li, Yangbo He, Zhihui Liu

**Affiliations:** ^1^ Integrated Agricultural Development Research Institute Guizhou Provincial Academy of Agricultural Sciences Guiyang China; ^2^ Guizhou Jinnong Irradiation Technology Co., Ltd. Guiyang China

**Keywords:** chicken meat, physicochemical properties, quality, γ‐Irradiation

## Abstract

Irradiation has become a mature processing approach for the quality control of many agricultural products. The effects of gamma irradiation at four different doses (2, 4, 6, and 8 kGy) on microbial quality, Hunter's parameter, lipid oxidation, hydrolyzed amino acids (HAAs), and flavor in spicy chicken were investigated. After treatment, all samples were stored at 4°C. The microbiological results showed that the total viable bacteria (TVB) and total coliform count (TCC) were significantly decreased dose dependently. Values of Δa*, Δb*, and ΔE* of the irradiated specimens were lower compared with the control samples, whereas the ΔL* of the irradiated specimens was higher compared with the controls. The peroxide value (POV) was increased by dose augmentation. Contents of HAAs were gradually decreased in both irradiated and control groups. The odor was affected by both doses of irradiation and storage time. Hence, we conclude that irradiation at a dose of 4.0 kGy barely affected physicochemical properties during storage and extended the shelf life of spicy chicken. This approach could be an alternative to control the quality of spicy chicken during storage.

## Graphical abstract

In this manuscript, we report the influence of gamma irradiation on microbial quality, Hunter's parameter, lipid oxidation, hydrolyzed amino acids, and flavor change. The results from our study showed that 4 kGy gamma irradiation could inhibit the growth of microorganisms and prolong shelf life, and had minor negative effects on the physicochemical properties of spicy chicken during storage.

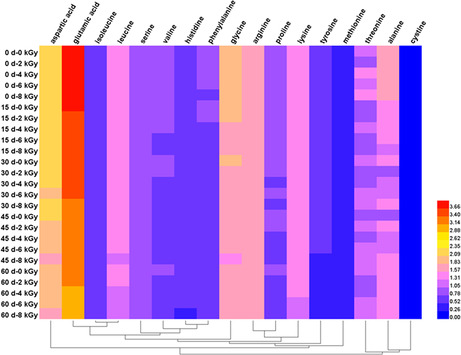

## 1 INTRODUCTION

Spicy chicken is a traditional delicacy in Guizhou Province of China due to its long history and rich nutritional value. The raw materials are chicken meat, red pepper, and chili oil. Its processing technology has been listed as a provincial‐level intangible cultural heritage. From the perspective of nutrition, chicken meat is characterized by high protein, low cholesterol, and low fat compared with pork, beef, and mutton. It also contains high contents of minerals and unsaturated fatty acids, which are widely favored by consumers (Hassanzadeh et al., 2017). Nutrient‐dense meat also provides good growth conditions for various pathogenic microorganisms, such as *Salmonella* spp., *Escherichia coli*, *Listeria monocytogenes*, *Streptococcus*, and *Micrococcus*, and hence the control of meat pathogens is a vital safety issue (Muhammad et al., 2019). In order to ensure the shelf life, it is necessary to add a variety of preservatives and adopt high‐temperature sterilization in the current production process of spicy chicken, which will destroy the taste and flavor. Color, flavor, sense perception, and appearance are essential properties that affect meat quality and economic value (Paula et al., 2019). Therefore, a proper sterilization method can prolong the shelf life of the product and retain the original quality of the product to the greatest extent. Irradiation technology is a processing method that uses high‐energy photons or charged particles to kill microorganisms and pests in food. As a typical nonthermal sterilization technology, it plays an increasingly important role in food safety, preservation, and storage (Amiri et al., 2019). The United Nations Food and Agriculture Organization (FAO), the World Health Organization (WHO), and the International Atomic Energy Agency (IAEA) confirmed in 1980 based on a large number of toxicological studies that any food irradiated below 10 kGy would not produce toxicological hazards (Amiri et al., 2019; Chen et al., 2016). China has also carried out many animal and human experiments on the safety of irradiated food (Li et al., 2015). It has been proved that irradiated food is harmless to the human body (Ravindran & Jaiswal, 2019). Chen et al. (2012) have investigated food irradiation in 133 food samples (33 kinds of seasonings, 20 kinds of dehydrated vegetables, 64 kinds of dehydrated dried fruits, and 16 kinds of pollens) purchased from the Chinese market. The results show that 30 samples are irradiated, accounting for 22.6% of the total samples. Irradiation technology has been widely used commercially, and more and more foods have been sterilized and kept fresh by irradiation. Nowadays, the application of irradiation technology predominates in the food industry, with more than 200 species of irradiated foods being approved for marketing by 50 countries (Cutrubinis et al., 2007; Li et al., 2019). Irradiation technology has been applied to improve the microbial quality of chicken meat, ensure its safety, and extend its shelf life with no adverse changes or deterioration (Abdeldaiem, 2014; Fallah et al., 2010; Rao et al., 2005). However, to our knowledge, no study has been conducted on the qualities of spicy chicken treated by irradiation of ^60^ Co γ‐ray. In the present study, we aimed to evaluate the effect of γ‐ray irradiation on microbiological, color, peroxide value (POV), amino acids, and odor of spicy chicken during storage.

## 2 MATERIALS AND METHODS

### 2.1 Sample preparation and irradiation treatment

The spicy chicken was provided by Guizhou Longshan Xiangfang Food Co, Ltd. Samples were divided into five groups with different doses of irradiation (2.0 , 4.0 , 6.0 , and 8.0 kGy), and nonirradiated (0 kGy) sample served as the control group. The spicy chicken of each group was vacuum packaged in polyethylene bags, and each bag contained 60 g of spicy chicken. All samples except for the control group were sealed in a corrugated case for ^60^Co γ‐irradiation at room temperature (25 ± 2 °C, RH 70%–75%), which was performed at Guizhou Jinnong Irradiation Technology Co., Ltd. The average dose rate of the irradiation facility was 0.25 kGy/h. After irradiation, samples were stored in a refrigerator (4 ± 2°C) for further analyses. The actual absorbed dose was determined by JJF 1028–91 (National Metrological Technical Code of the People's Republic of China: Standard Method for Using the Silver Dichromate Dosimeter to Measure γ‐Ray Absorbed Dose in Water). Three dichromate dosimeters were placed in the front, rear, and center of the corrugated case for each irradiation group.

### 2.2 Microbiological evaluation

The microbial counts of the total viable bacteria (TVB) were determined following the National Standard of the People's Republic of China GB 4789.2–2016. The total coliform count (TCC) was determined by the method described by GB 4789.3–2016 using the most probable number (MPN) index. *Salmonella* spp. was tested by the method described by GB 4789.4–2016. The counts of *Listeria monocytogenes* were determined following GB 4789.30. The counts of *Staphylococcus aureus* were determined using the second method in GB 4789.10–2016. Samples were collected at 0, 15, 30, 45, and 60 days after irradiation treatment.

### 2.3 Hunter's color measurement

The appearance of spicy chicken depends on the chili oil in the spicy chicken packages. Color values were determined on the surface of spicy chicken packages by using a high‐quality colorimeter NR 200 (3NH Technology Co., Ltd). The experiment was repeated three times, and each replicate included three bags. Samples were collected at 0, 15, 30, 45, and 60 days after irradiation. The standard white plate (*L* = 90.60, *a* = 0.23, *b* = −4.02) was used to calibrate. The color difference (ΔE*) was calculated from the following equation:

$$\Delta E^{*}=\sqrt{{\left(\Delta L^{*}\right)}^{2}+{\left(\Delta a^{*}\right)}^{2}+{\left(\Delta b^{*}\right)}^{2}}$$

### 2.4 POV determination

Briefly, 60 g of chicken meat from three bags was ground in a blender, followed by the addition of 120‐ml petroleum ether (boiling range: 30–60°C). The mixture was shaken well, fully mixed, stood, and extracted for 12 h, followed by filtration through a funnel containing anhydrous sodium sulfate. Next, the filtrate was collected and evaporated under reduced pressure with a rotary evaporator in a water bath at 40°C, and the residue was the sample to be tested. Next, 2.0000 g of sample was accurately weighed and put into a 250‐ml iodine measuring bottle, and then 30 ml of chloroform/glacial acetic acid mixture was added. The mixture was shaken gently to dissolve the sample completely. Subsequently, 1.00 ml of saturated potassium iodide solution was accurately added, and the bottle cap was plugged. The bottle was gently shaken for 0.5 min and placed in the dark for 3 min. Then, 100 ml of water was added to the bottle. The precipitated iodine was immediately titrated with sodium thiosulfate standard solution (0.002 mol/L). When light yellow color appeared, 1‐ml starch indicator was added, and the solution was continuously titrated until the blue of the solution disappeared as the endpoint.

### 2.5 Amino acid analysis

#### 2.5.1 Sample hydrolysis

Briefly, 0.2 g of the sample was accurately weighed into a 50‐ml hydrolysis tube, followed by adding 20‐ml HCl. The mixture was hydrolyzed in an oven at 110°C for 22 h. After cooling, the sample was transferred to a 25‐ml colorimetric tube for constant volume.

#### 2.5.2 Derivatization

Briefly, 50 μl of the sample was accurately taken and put into a 4‐ml centrifuge tube. The tube was put into a vacuum drying oven at 60°C for 2 h, the centrifuge tube was filled with nitrogen, and then 50 μl of derivatization reagent (ethanol: phenyl isothiocyanate: water: triethylamine = 7:1:1:1) was accurately added. The solution was prepared just before use. It was filled with nitrogen during preparation and derivatized at room temperature for 30 min. Subsequently, 0.45 ml of mobile phase was added, and the mixture was mixed well and filtered through 0.25‐μm polyester filters.

#### 2.5.3 Determination

The amino acids of chicken meat were determined by high‐performance liquid chromatography (HPLC) using Agilent 1260 HPLC (Agilent Technologies) system equipped with a ZORBAX 83 SB‐C18 column (250 × 4.6 mm, 5 μm). The oven was operated at 40°C, and the sample injection volume was 10 μl. The mobile phase was composed of 97% of 0.1 M phosphate buffer solution (pH = 6.5) (A) and 5% methanol (B).

### 2.6 Electronic nose

The electric noise was determined using the commercial PEN3 Portable Electronic Nose (Airsense Analytics Inc.) as previously described (2019). The machine was composed of 10 metal oxide semiconductor sensors (W1C, W5S, W3C, W6S, W5C, W1S, W1W, W2S, W2W, and W3S). Each sensor featured different sensitivities to various chemical components (Table 1). Briefly, 3 g of chicken meat was detected by the E‐nose for each group. The sample in the headspace bottle was sealed, whereas the headspace generation time reached 120 min. The measurement and flushing time were 120 s and 5 s, respectively. Three replicate samples were prepared for each experiment. Response values of E‐nose were recorded and analyzed by principal component analysis (PCA), linear discriminant analysis (LDA), and loading analysis (LA).

**TABLE 1:** Sensors used in E‐nose.

Number in array	Sensor name	General description	Limit of detection
1	W1C	aroma component	Methylbenzene, 10 ml*m^−1^
2	W5S	Very sensitive to oxynitride	NO_2_,1 ml*m^−1^
3	W3C	ammonium hydroxide, sensitive to aroma component	Benzene, 10 ml*m^−1^
4	W6S	Mainly hydrogen, selectively	H_2_, 100 ml*m^−1^
5	W5C	Alkanes, aromatic compounds	Propane, 1 ml*m^−1^
6	W1S	Sensitive to methane	CH_4_, 100 ml*m^−1^
7	W1W	Sensitive to sulfide	H_2_S, 1 ml*m^−1^
8	W2S	Sensitive to ethyl alcohol	CO, 100 ml*m^−1^
9	W2W	Aromatics compounds, sensitive to sulfur organic compounds	H_2_S, 1 ml*m^−1^
10	W3S	Sensitive to alkane	CH_4_, 10 ml*m^−1^

### 2.7 Statistical analysis

The data were presented as mean ± standard deviation (SD), and the significant differences among the groups were analyzed by SPSS version 22.0 software (SPSS, Inc.) with 95% confidence. Data were analyzed by one‐way analysis of variance (ANOVA) to test the treatment effect. E‐nose data were processed by Win Musterversion 1.6 (Airsense Analytics Inc.). Mean separations were performed by Duncan's multiple range tests. Differences at *p* < .05 were considered statistically significant.

## 3 RESULTS AND DISCUSSION

### 3.1 Dosimetry

The actual absorbed dose may be different owing to the different positions of the sample in the basket. Therefore, the actual absorbed dose was measured by a solver dichromate dosimeter. The actual absorbed dose of four irradiated groups of the spicy chicken samples was 1.93, 4.12, 6.04, and 7.96.

### 3.2 Microbiological test of irradiated chicken meat

TVB, TCC, *Salmonella* spp., *S. aureus*, and *L. monocytogenes* were measured, and Table 2 presents the results. *Salmonella* spp., *S. aureus*, and *L. monocytogenes* were detected neither in the control group (0 kGy) nor in four irradiated groups (2, 4, 6, and 8 kGy), which could be probably attributed to the fact that the sample was boiled and fried at a high temperature, eliminating those three pathogenic bacteria. Table 2 shows that TVB in the control group ranged from 4.20 to 5.03, which ranged from 3.85 to 4.17 in the 2 kGy group, from 3.17 to 2.74 in the 4 kGy group, from 2.64 to 1.95 in the 6 kGy group, and from 2.15 to 1.00 in the 8 kGy group. TVB in all irradiated groups was significantly (*p* < .05) decreased with the extended storage time compared with the control group. On the contrary, TVB in the 0 kGy and 2 kGy groups was increased during the whole storage time, and TVB of the 4 kGy group was gradually decreased until the 13th day and then increased in the rest storage period. TCC in four irradiated groups was always lower than 3.0 MPN/g during the whole storage period, and this value in the nonirradiated group was increased at 45 and 60 days. The above results indicated that the irradiation dose greater than 6 KGy could inhibit the propagation of microorganisms in the spicy chicken, total coliforms seemed to be more sensitive to irradiation, and a dose of 2 kGy could control the growth of coliforms. A similar result has been found by Wellington et al. (2014) that low doses of gamma radiation can efficiently control pathogens in chicken meat. Moreover, gamma irradiation is the earliest irradiation method used in meat processing, which can not only control the growth of foodborne microorganisms but also improve the quality of meat (Hassanzadeh et al., 2017). Coliform is an indicator of fecal pollution. It exists in various environments and can be easily polluted in food processing. The effects of disinfection by radiation depend on the energy source, dose rate, and absorbed dose (Kyung et al., 2019). Xavier et al. (2016) have demonstrated that even 1.5 kGy of gamma irradiation is sufficient to eliminate enteropathogen from fresh chicken meat. The mechanism underlying the germicidal effect of irradiation is the ionization energy absorbed by DNA molecules. The phosphodiester bond in the DNA double helix structure is broken, resulting in the inability of cells to replicate themselves.

**TABLE 2:** Irradiation on TVB and TCC of spicy chicken.

Time (d)	Dose (kGy)	Total viable bacteria (log CFU × g^−1^)	Total coliform counts (MPN × g^−1^)
0	0	4.20 ± 0.05 a	<3.0
	2	3.85 ± 0.11 b	<3.0
	4	3.17 ± 0.11 c	<3.0
	6	2.64 ± 0.07 d	<3.0
	8	2.15 ± 0.08 e	<3.0
15	0	4.35 ± 0.09 a	<3.0
	2	3.27 ± 0.06 b	<3.0
	4	2.65 ± 0.17 c	<3.0
	6	2.39 ± 0.09 d	<3.0
	8	1.70 ± 0.09 e	<3.0
30	0	4.48 ± 0.06 a	<3.0
	2	3.38 ± 0.08 b	<3.0
	4	2.30 ± 0.10 c	<3.0
	6	2.20 ± 0.07 c	<3.0
	8	1.48 ± 0.07 d	<3.0
45	0	4.81 ± 0.11 a	6.16 ± 0.06
	2	3.91 ± 0.06 b	<3.0
	4	2.51 ± 0.12 c	<3.0
	6	2.11 ± 0.13 d	<3.0
	8	1.30 ± 0.11 e	<3.0
60	0	5.03 ± 0.10 a	9.8 ± 2.07
	2	4.17 ± 0.09 b	<3.0
	4	2.74 ± 0.12 c	<3.0
	6	1.95 ± 0.05 d	<3.0
	8	1.00 ± 0.09 e	<3.0

*Note*: Data represent mean values ± standard deviation (*n* = 3). Values with different letters at each time point are significantly different according to Duncan's multiple range test (*p* < .05).

### 3.3 Hunter's color

**FIGURE 1:**
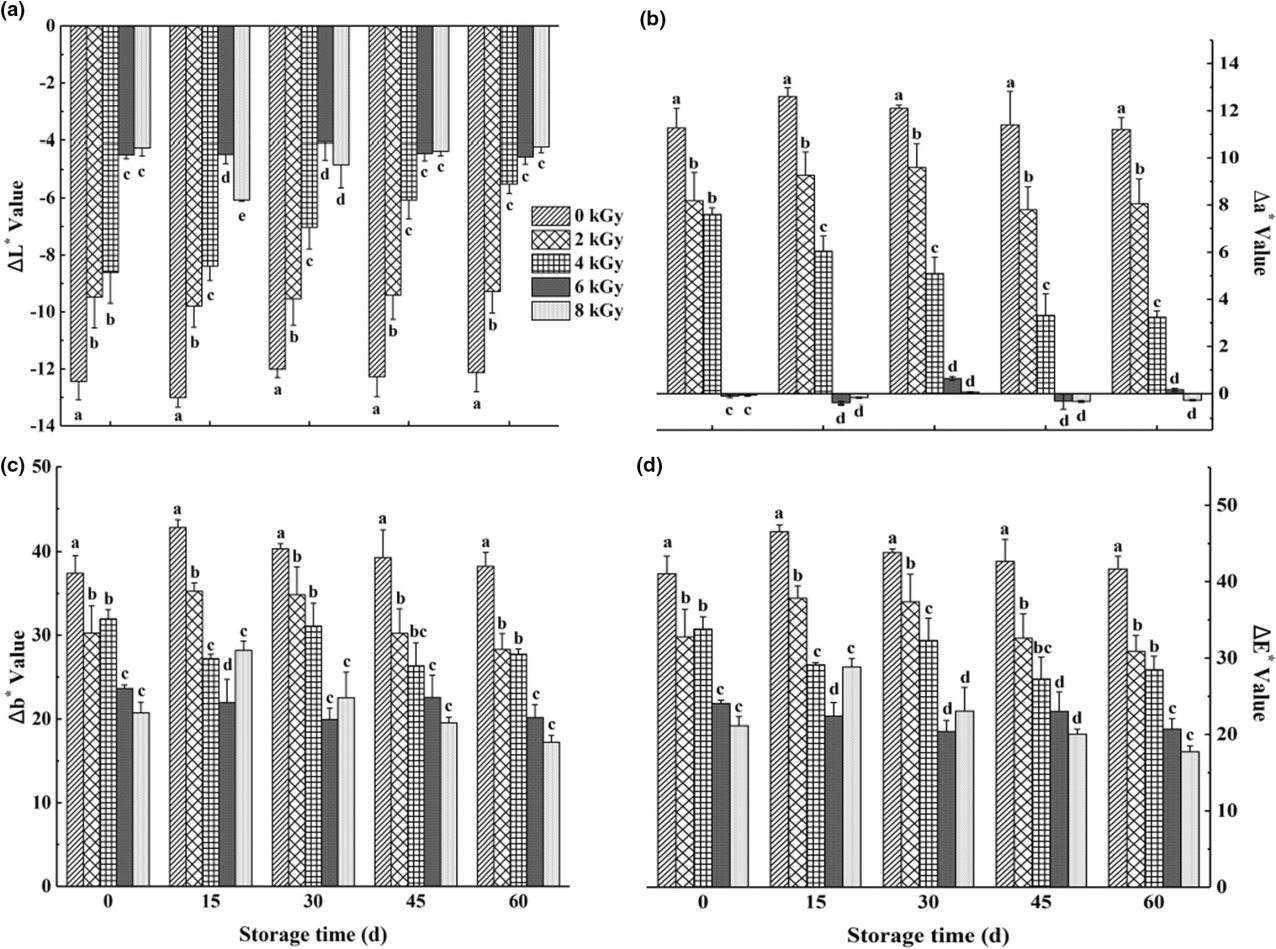
Effect of different doses of gamma ray on Hunter's color values of spicy chicken during storage. a, B, c, and D indicate values of ΔL*, Δa*, Δb*, and ΔE*, respectively. Each column represents the mean of three replicates. Bars represent standard deviations of the means. Columns with different letters at each time point are significantly different according to Duncan's multiple range test (*p* < .05).

Spicy chicken contains a lot of chili oil, which is rich in red carotenoids, including capsanthin. Capsanthin is the primary color substance of mature pepper, which is primarily present mostly in both the free form and the esterified form with fatty acids. The values of ΔL*, Δa*, and Δb* represent lightness, redness, and yellowness, respectively. The color of spicy chicken was obviously affected by the four doses of irradiation (Figure 1). The ΔL* values of the four irradiated groups were significantly higher compared with the nonirradiated group (*p* < .05). The change of ΔL * was not apparent in the 0 , 2 , and 6 kGy groups during the whole storage period.

In contrast, this value in the 4 kGy group was gradually increased, while it reached the minimum of 8 kGy on the 15th day after irradiation and then was gradually increased. At the end of storage, the value of ΔL* of the 4, 6, and 8 kGy groups was significantly higher compared with the control group and 2 kGy group. The value of Δa* was significantly (*p* < .05) decreased during the whole storage period compared with the control group. Moreover, the value of Δa* in the 6 and 8 kGy groups was dramatically decreased, which remained at low levels during the storage time (Figure 1b). Irradiation treatment significantly decreased the value of Δb*. At the end of storage, the Δb* value was gradually reduced with the increase in irradiation dose (Figure 1c). For the value of ΔE* in spicy chicken, it was significantly (*p* < .05) decreased during the whole storage period by irradiation. The value of ΔE* in the 0, 2, and 8 kGy groups was gradually increased. It peaked on day 15, and then the value of ΔE* decreased slightly.

Moreover, no apparent fluctuation was observed in the 4 and 6 kGy groups (Figure 1d). Ding et al. (2015) have revealed the underlying mechanism of capsanthin fading, which is attributed to direct oxidation or addition reactions in the presence of reactive oxygen species (ROS). Besides, water molecules in chicken meat produced ROS when irradiated, and the double bonds and carbonyl groups in capsanthin reacted with ROS. However, the capsanthin in the dried red chili powder was not changed obviously after gamma irradiation. In our present study, the chili oil in spicy chicken samples faded, which might be attributed to the fact that the irradiation accelerates the automatic oxidation process of unsaturated fatty acids in the oil. Such a process produces many oxygen‐containing products, such as hydroperoxides and carbonyl compounds, which can react with capsanthin and result in fading (Jung et al., 2015).

### 3.4 POV

Chicken meat contains relatively high amounts of unsaturated fatty acids, making chicken meat considerably susceptible to deterioration caused by oxidation processes (Xiao et al., 2011). Peroxides are reported as the primary products of lipid oxidation. POV signifies the number of peroxides, which is an essential index for evaluating the storage stability of oils and fats (Gray et al., 1978). POV is an important quality indicator in irradiated meat samples, representing the degree of lipid damage caused by irradiation (Nisar et al., 2019). Irradiation generates free radicals that can induce lipid peroxidation (Zheng et al., 2022).

**FIGURE 2:**
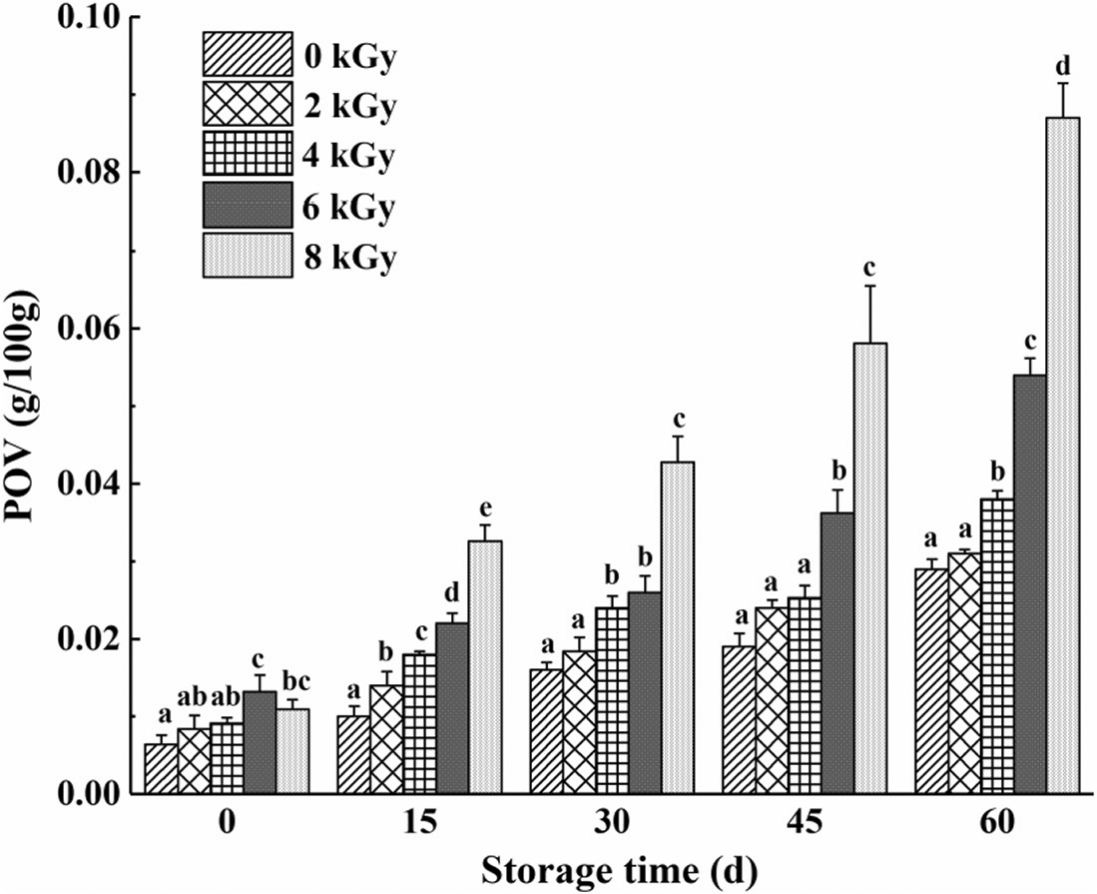
Effect of different doses of gamma ray on the lipid oxidation levels of spicy chicken during storage. Each column represents the mean of three replicates (each of 20 fruits). Bars represent standard deviations of the means. Columns with different letters at each time point are significantly different according to Duncan's multiple range test (*p* < .05).

Oxidative degradation of lipids from the spicy chicken was assessed by POV values (Figure 2). POV in the irradiated groups was increased dose dependently, which was significantly enhanced after 15 days of storage in the 6 and 8 kGy groups. The POV in the 2 kGy group was not significantly altered except for the 15th day after treatment. The POV value in the 4 kGy group was significantly (*p* < .05) increased except for the 0 and 45th days after treatment compared with the nonirradiated group. At the end of storage, the POV value in chicken meat treated with 4, 6, and 8 kGy irradiation was higher compared with the control group, which was 31.03%, 86.20%, and 200.00%, respectively.

As one of the critical factors, lipid oxidation also affects volatile compounds in foods. Free radical chain reactions cause oxidative changes in food lipids. The degradation of hydroperoxides, the main product of lipid oxidation, creates a complex mixture of low‐molecular‐weight compounds with distinct flavor characteristics (Gray and Monahan, 1992).

### 3.5 Hydrolyzed amino acids (HAAs)

**FIGURE 3:**
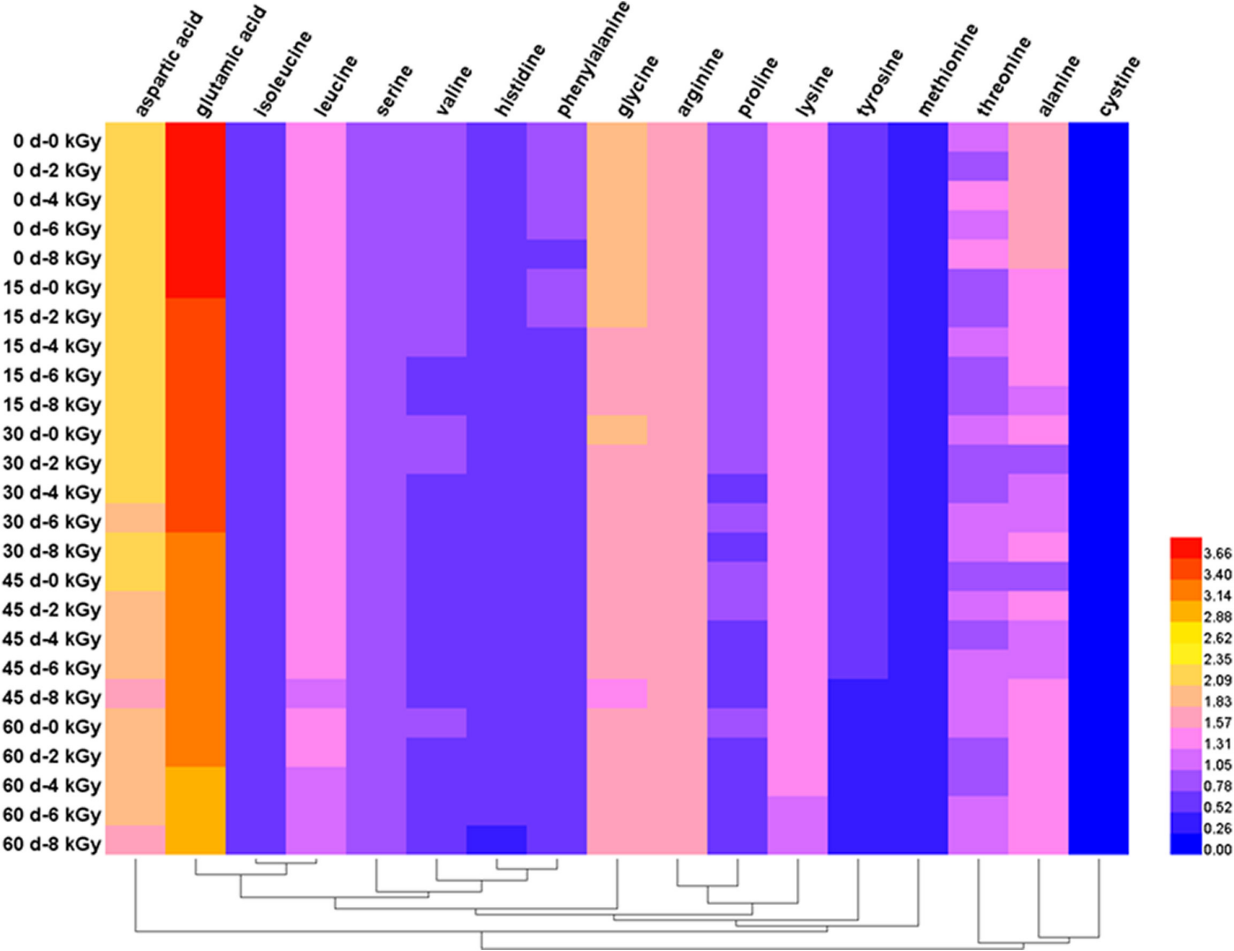
Heatmap of contents of HAAs (g/100 g) in irradiated spicy chicken meat.

A heatmap was illustrated by Heml software (version 1.0), showing the variations in the contents of HAAs in spicy chicken irradiated at four different doses (Figure 3). The content of each component in the figure is represented by different colors, in which the darker the red, the higher the content, and the deeper the blue, the lower the content. The samples irradiated at different irradiation doses were clustered into two clusters. The first cluster contained threonine, alanine, and cystine, which all belonged to aromatic amino acids. The second cluster contained aliphatic amino acids (aspartic acid, glutamic acid, serine, glycine, arginine, threonine, alanine, proline, valine, methionine, cystine, isoleucine, leucine, and lysine).

Table 3 shows that the HAA contents (g/100 g) were determined in spicy chicken meat after being irradiated at four doses and stored for 60 days. All HAAs were decreased after irradiation and storage. Aspartic acid, glutamic acid, serine, arginine, proline, valine, methionine, isoleucine, leucine, lysine, and histidine were not significantly changed at different irradiation doses with the same sampling time. The contents of total HAAs were 18.62, 18.25, 17.95, and 17.70 in the 2, 4, 6, and 8 kGy groups at the end of storage time (60th day), respectively, which were decreased by 4.36%, 6.23%, 7.76%, and 9.07% compared with the control group, respectively. The effects of irradiation on aliphatic and aromatic amino acids are different (Jayathilakan & Sultana, 2018). The aromatic amino acids, including histidine, phenylalanine, and tyrosine, in all irradiated groups were decreased by 4.49%, 9.03%, 11.39%, and 13.34% compared with the control group at the end of storage, respectively. In contrast, the aliphatic amino acids were decreased by 4.35%, 5.95%, 7.39%, and 8.63%, respectively. The result demonstrated that the aromatic amino acids were more sensitive than aliphatic amino acids. Nisar et al. (2019) have found that irradiation at 1.5 and 3.0 kGy can reduce the contents of HAAs in chicken meat, which is consistent with our findings. Guillén‐Casla et al. (2010) have also reported a decrement in tryptophan, phenylalanine, and tyrosine components of minced meat when irradiated at 1–8 kGy. Such reduction of amino acids in irradiated chicken meat could be attributed to the peroxy radicals generated by irradiation. Ahn et al. (2000) have shown that the radiolytic breakdown of sulfur‐containing amino acids significantly contributes to off‐odor in irradiated meat.

**TABLE 3:** Compositions of hydrolyzed amino acids (g/100 g) in irradiated spicy chicken.

Dose (kGy)	Hydrolyzed amino acids																
	ASP	GLU	SER	GLY	ARG	THR	ALA	PRO	VAL	MET	CYS	ILEU	LEU	LYS	HIS	PHE	TYR
0 d																	
0	2.25 ± 0.14a	3.82 ± 0.22a	0.99 ± 0.08a	1.94 ± 0.13a	1.74 ± 0.12a	1.05 ± 0.06bc	1.64 ± 0.10ab	0.89 ± 0.04a	0.86 ± 0.06a	0.50 ± 0.02a	0.11 ± 0.01a	0.76 ± 0.06a	1.45 ± 0.07a	1.42 ± 0.09a	0.63 ± 0.04a	0.85 ± 0.04a	0.61 ± 0.03a
2	2.18 ± 0.10a	3.80 ± 0.16a	0.98 ± 0.11a	1.94 ± 0.17a	1.74 ± 0.07a	0.94 ± 0.04c	1.73 ± 0.08a	0.86 ± 0.07a	0.84 ± 0.08a	0.47 ± 0.03a	0.08 ± 0.00b	0.73 ± 0.03a	1.43 ± 0.05a	1.46 ± 0.07a	0.62 ± 0.03a	0.83 ± 0.06ab	0.59 ± 0.04a
4	2.15 ± 0.06a	3.81 ± 0.19a	0.96 ± 0.06a	1.93 ± 0.12a	1.72 ± 0.08a	1.33 ± 0.04a	1.74 ± 0.09a	0.85 ± 0.07a	0.84 ± 0.05a	0.46 ± 0.03a	0.07 ± 0.00b	0.74 ± 0.05a	1.45 ± 0.11a	1.39 ± 0.11a	0.61 ± 0.03a	0.80 ± 0.06ab	0.58 ± 0.03a
6	2.27 ± 0.08a	3.92 ± 0.31a	0.96 ± 0.07a	1.92 ± 0.07a	1.72 ± 0.10a	1.19 ± 0.05ab	1.58 ± 0.11b	0.85 ± 0.06a	0.84 ± 0.06a	0.45 ± 0.04a	0.05 ± 0.00c	0.72 ± 0.04a	1.42 ± 0.10a	1.38 ± 0.08a	0.61 ± 0.02a	0.78 ± 0.05ab	0.58 ± 0.02a
8	2.23 ± 0.11a	3.71 ± 0.21a	0.95 ± 0.09a	1.91 ± 0.10a	1.70 ± 0.06a	1.32 ± 0.08a	1.73 ± 0.06a	0.82 ± 0.05a	0.82 ± 0.07a	0.42 ± 0.03a	0.03 ± 0.00d	0.72 ± 0.06a	1.41 ± 0.08a	1.37 ± 0.07a	0.57 ± 0.05a	0.75 ± 0.03b	0.57 ± 0.04a
15 d																	
0	2.21 ± 0.17a	3.74 ± 0.33a	0.96 ± 0.04a	1.93 ± 0.13a	1.72 ± 0.10a	1.03 ± 0.08ab	1.39 ± 0.06ab	0.87 ± 0.04a	0.82 ± 0.06a	0.48 ± 0.02a	ND^a^	0.73 ± 0.04a	1.43 ± 0.09a	1.41 ± 0.12a	0.61 ± 0.03a	0.82 ± 0.06a	0.59 ± 0.02a
2	2.18 ± 0.14a	3.64 ± 0.25a	0.96 ± 0.06a	1.88 ± 0.16ab	1.71 ± 0.09a	0.90 ± 0.05bc	1.36 ± 0.09ab	0.85 ± 0.06a	0.80 ± 0.04a	0.48 ± 0.04a	ND	0.72 ± 0.06a	1.40 ± 0.06a	1.39 ± 0.07a	0.60 ± 0.04a	0.79 ± 0.05ab	0.57 ± 0.04a
4	2.20 ± 0.08a	3.49 ± 0.21a	0.93 ± 0.07a	1.71 ± 0.11abc	1.68 ± 0.14a	1.17 ± 0.10a	1.37 ± 0.07ab	0.83 ± 0.03a	0.79 ± 0.05a	0.47 ± 0.02a	ND	0.72 ± 0.02a	1.40 ± 0.10a	1.39 ± 0.08a	0.57 ± 0.02a	0.78 ± 0.05abc	0.55 ± 0.02a
6	2.16 ± 0.18a	3.46 ± 0.16a	0.94 ± 0.08a	1.68 ± 0.08bc	1.68 ± 0.08a	1.00 ± 0.08bc	1.46 ± 0.11a	0.83 ± 0.03a	0.77 ± 0.03a	0.45 ± 0.01a	ND	0.70 ± 0.03a	1.38 ± 0.07a	1.38 ± 0.11a	0.58 ± 0.05a	0.74 ± 0.02bc	0.53 ± 0.03a
8	2.13 ± 0.11a	3.48 ± 0.28a	0.90 ± 0.06a	1.62 ± 0.14c	1.67 ± 0.05a	0.87 ± 0.06c	1.27 ± 0.06b	0.83 ± 0.04a	0.74 ± 0.05a	0.42 ± 0.02a	ND	0.69 ± 0.05a	1.35 ± 0.04a	1.37 ± 0.06a	0.56 ± 0.01a	0.71 ± 0.03c	0.53 ± 0.02a
30 d																	
0	2.11 ± 0.12a	3.45 ± 0.20a	0.95 ± 0.07a	1.85 ± 0.11a	1.72 ± 0.06a	1.15 ± 0.04a	1.31 ± 0.07ab	0.84 ± 0.03a	0.80 ± 0.05a	0.48 ± 0.03a	ND	0.71 ± 0.03a	1.39 ± 0.08a	1.40 ± 0.04a	0.59 ± 0.02a	0.77 ± 0.05a	0.57 ± 0.01a
2	2.14 ± 0.06a	3.43 ± 0.16a	0.94 ± 0.04a	1.75 ± 0.07ab	1.72 ± 0.07a	0.89 ± 0.03a	1.00 ± 0.05c	0.81 ± 0.05a	0.79 ± 0.03a	0.46 ± 0.02a	ND	0.70 ± 0.03a	1.38 ± 0.06a	1.39 ± 0.08a	0.58 ± 0.02a	0.76 ± 0.03a	0.57 ± 0.01a
4	2.09 ± 0.14a	3.40 ± 0.18a	0.93 ± 0.05a	1.71 ± 0.10ab	1.66 ± 0.10a	0.90 ± 0.04a	1.18 ± 0.04b	0.78 ± 0.03a	0.77 ± 0.04a	0.45 ± 0.04a	ND	0.68 ± 0.05a	1.38 ± 0.10a	1.37 ± 0.06a	0.56 ± 0.03a	0.73 ± 0.05a	0.56 ± 0.04a
6	2.03 ± 0.18a	3.43 ± 0.24a	0.89 ± 0.06a	1.69 ± 0.13ab	1.67 ± 0.13a	1.11 ± 0.06a	1.26 ± 0.07ab	0.79 ± 0.04a	0.75 ± 0.03a	0.43 ± 0.01a	ND	0.66 ± 0.02a	1.37 ± 0.05a	1.36 ± 0.06a	0.55 ± 0.01a	0.73 ± 0.01a	0.54 ± 0.02a
8	2.09 ± 0.17a	3.33 ± 0.23a	0.87 ± 0.04a	1.60 ± 0.08b	1.65 ± 0.09a	1.14 ± 0.05a	1.33 ± 0.10a	0.76 ± 0.03a	0.74 ± 0.04a	0.42 ± 0.02a	ND	0.66 ± 0.01a	1.34 ± 0.09a	1.34 ± 0.11a	0.54 ± 0.03a	0.69 ± 0.03a	0.52 ± 0.02a
45 d																	
0	2.11 ± 0.11a	3.39 ± 0.24a	0.92 ± 0.06a	1.78 ± 0.04a	1.71 ± 0.06a	1.06 ± 0.03ab	1.36 ± 0.08a	0.82 ± 0.05a	0.77 ± 0.05a	0.46 ± 0.02a	ND	0.67 ± 0.02a	1.37 ± 0.10a	1.38 ± 0.07a	0.58 ± 0.02a	0.74 ± 0.05ab	0.54 ± 0.02a
2	2.04 ± 0.16a	3.30 ± 0.18a	0.92 ± 0.04a	1.73 ± 0.09a	1.68 ± 0.07a	1.25 ± 0.11a	1.41 ± 0.07a	0.78 ± 0.03a	0.77 ± 0.04a	0.46 ± 0.02a	ND	0.66 ± 0.05a	1.34 ± 0.07a	1.36 ± 0.11a	0.57 ± 0.02a	0.76 ± 0.05a	0.53 ± 0.02a
4	2.07 ± 0.14a	3.27 ± 0.11a	0.93 ± 0.03a	1.73 ± 0.05a	1.66 ± 0.12a	0.99 ± 0.06b	1.23 ± 0.10a	0.76 ± 0.03a	0.75 ± 0.04a	0.43 ± 0.03a	ND	0.63 ± 0.03a	1.34 ± 0.05a	1.34 ± 0.08a	0.55 ± 0.04a	0.72 ± 0.04ab	0.54 ± 0.01a
6	2.04 ± 0.07a	3.27 ± 0.20a	0.90 ± 0.04a	1.66 ± 0.11a	1.64 ± 0.10a	1.15 ± 0.05ab	1.26 ± 0.08a	0.75 ± 0.05a	0.73 ± 0.06a	0.40 ± 0.02a	ND	0.64 ± 0.03a	1.33 ± 0.05a	1.32 ± 0.06a	0.56 ± 0.03a	0.69 ± 0.03bc	0.52 ± 0.01a
8	1.81 ± 0.12a	3.17 ± 0.15a	0.88 ± 0.05a	1.54 ± 0.06a	1.63 ± 0.05a	1.21 ± 0.07a	1.39 ± 0.07a	0.73 ± 0.05a	0.71 ± 0.03a	0.38 ± 0.01a	ND	0.62 ± 0.02a	1.30 ± 0.08a	1.33 ± 0.11a	0.52 ± 0.03a	0.66 ± 0.03c	0.50 ± 0.03a
60 d																	
0	1.88 ± 0.04a	3.31 ± 0.16a	0.91 ± 0.05a	1.70 ± 0.13a	1.69 ± 0.09a	1.29 ± 0.10a	1.53 ± 0.06a	0.79 ± 0.03a	0.79 ± 0.02a	0.43 ± 0.02a	ND	0.65 ± 0.02a	1.33 ± 0.04a	1.36 ± 0.06a	0.58 ± 0.02a	0.74 ± 0.03a	0.48 ± 0.02a
2	1.88 ± 0.08a	3.24 ± 0.07a	0.89 ± 0.05a	1.67 ± 0.06a	1.66 ± 0.06a	0.96 ± 0.04c	1.38 ± 0.09a	0.74 ± 0.06a	0.76 ± 0.02a	0.40 ± 0.01a	ND	0.64 ± 0.03a	1.32 ± 0.07a	1.35 ± 0.09a	0.54 ± 0.02a	0.72 ± 0.02ab	0.45 ± 0.02ab
4	1.99 ± 0.13a	3.10 ± 0.21a	0.89 ± 0.03a	1.62 ± 0.08a	1.63 ± 0.12a	1.03 ± 0.07bc	1.32 ± 0.10a	0.67 ± 0.02a	0.74 ± 0.04a	0.40 ± 0.03a	ND	0.60 ± 0.01a	1.29 ± 0.11a	1.33 ± 0.05a	0.53 ± 0.04a	0.67 ± 0.02bc	0.43 ± 0.03bc
6	1.86 ± 0.06a	3.06 ± 0.13a	0.86 ± 0.06a	1.60 ± 0.08a	1.59 ± 0.07a	1.05 ± 0.07bc	1.40 ± 0.05a	0.64 ± 0.04a	0.71 ± 0.03a	0.39 ± 0.01a	ND	0.61 ± 0.03a	1.29 ± 0.07a	1.30 ± 0.04a	0.53 ± 0.01a	0.66 ± 0.04bc	0.40 ± 0.01c
8	1.69 ± 0.04a	3.03 ± 0.15a	0.82 ± 0.02a	1.57 ± 0.14a	1.60 ± 0.13a	1.19 ± 0.09ab	1.38 ± 0.06a	0.63 ± 0.04a	0.69 ± 0.02a	0.38 ± 0.02a	ND	0.59 ± 0.02a	1.27 ± 0.03a	1.31 ± 0.06a	0.51 ± 0.03a	0.63 ± 0.03c	0.41 ± 0.01c

*Note*: Data represent mean values ± standard deviation (*n* = 3). Values with different letters at each time point are significantly different according to Duncan's multiple range test (*p* < .05).

^a^ ND, not detected.

### 3.6 E‐nose

#### 3.6.1 PCA

**FIGURE 4:**
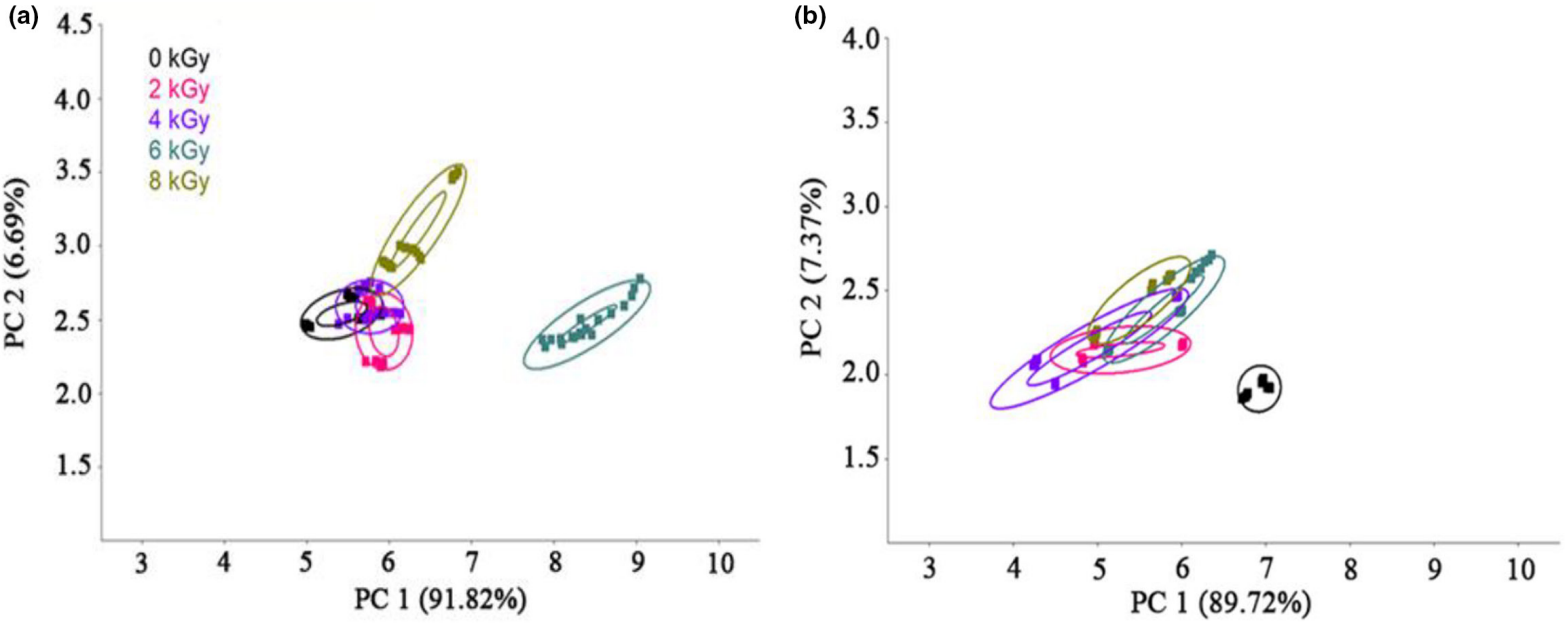
PCA plots of irradiated spicy chicken at different doses and storage time of 0 (a) and 60 days (b).

PCA was performed to profile the odor of irradiated spicy chicken at the beginning (0 day) and end (60 days) of storage (Figure 4). The first principal component (PC1) and the second principal component (PC2) accounted for 91.28% and 6.69% of the total variations, respectively. After 60 days of storage, PC1 and PC2 explained 89.72% and 7.37% of the total variance, respectively. The accumulative variance contribution rate of the two major components was larger than 90%, indicating that PC1 and PC2 covered the sample's prime information characteristics (Li et al., 2019). Clusters of 0 , 2 , and 4 kGy overlapped entirely, indicating that the flavor of chicken irradiated at these two doses was not distinguished from nonirradiated chicken. In contrast, clusters of 6 and 8 kGy were separated by the first two principal components. Moreover, the 6.0 kGy group exhibited a longer distance from the 0 kGy cluster compared with other doses, indicating that these two doses resulted in better discrimination than 0 kGy (Figure 4a). However, during 60 days of storage, four groups of irradiated chicken meat were separated from 0 kGy, showing a longer distance from 0 kGy based on PC1 (Figure 4b). Therefore, the irradiation dose remarkably affected the flavor of spicy chicken during the storage period.

#### 3.6.2 LDA

**FIGURE 5:**
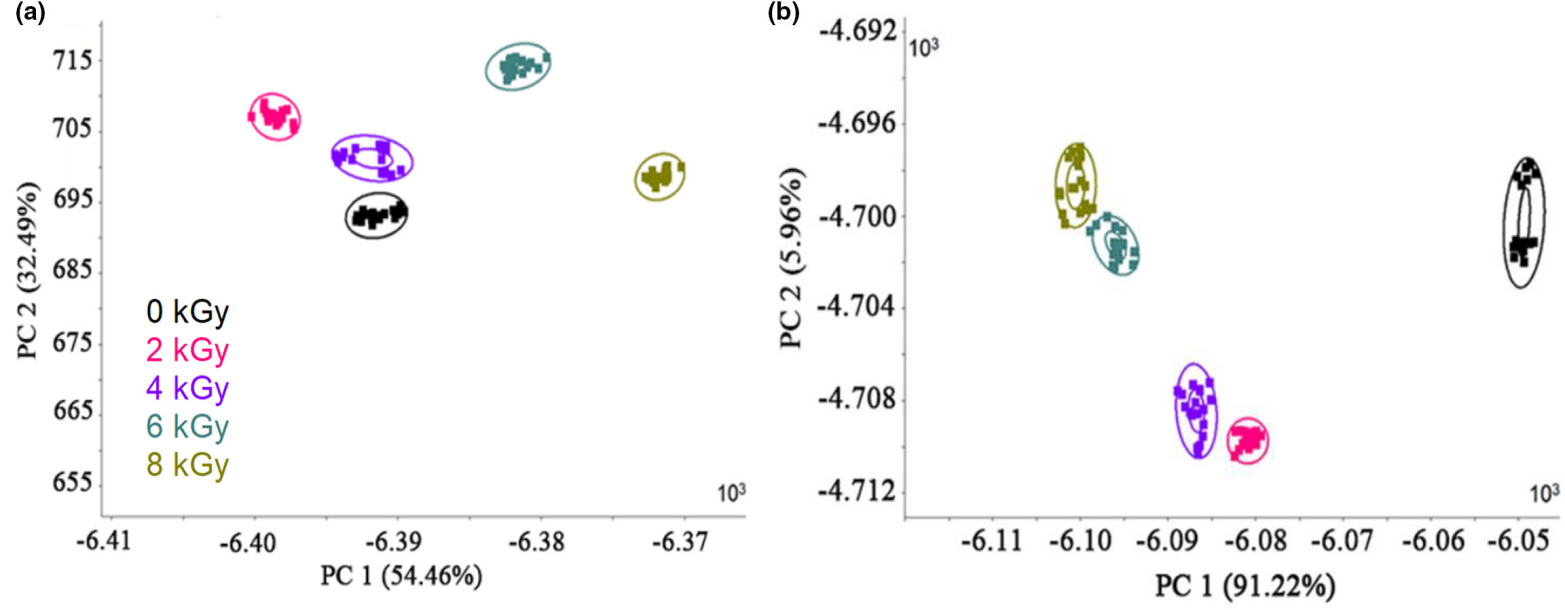
LDA plots of irradiated spicy chicken at different doses and storage time of 0 (a) and 60 days (b).

The LDA method aims to construct a linear transformation to achieve maximum class discrimination by a linear combination of data (Zhou, 2017). The response values of four different doses of irradiation and nonirradiated chicken meat at the beginning (0 day) (Figure 5a) and end of storage time (60th day) (Figure 5b) were determined. Five clusters were completely distinguished at 0 day. The distance of 2.0 and 4.0 kGy was close to 0 kGy based on PC1, while clusters of 6.0 and 8.0 kGy showed a longer distance from 0 kGy. The same results were observed at 60 days of storage time. After 60 days of storage, the distance of 2.0 and 4.0 kGy became longer than that at the beginning of the storage.

#### 3.6.3 LA

**FIGURE 6:**
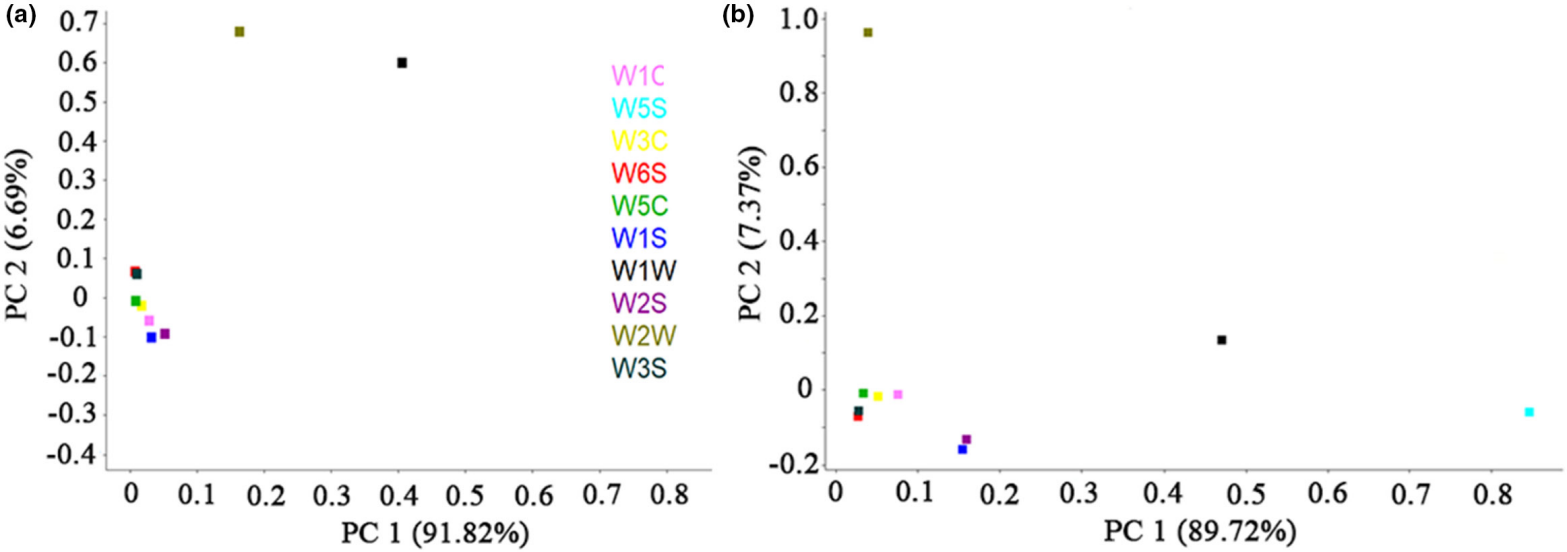
Loading plots of irradiated spicy chicken at different doses and storage time of 0 (a) and 60 days (b).

LA was conducted to identify the contribution of commonly used sensors responsible for distinction in PCA patterns (Zhang et al., 2007). The contribution of each metal oxide semiconductor sensor to the total response of the array could be judged by the location of the sensors in Figure 6. Sensors with loading parameters featuring a long distance to zero point indicate high contribution, whereas low values imply minor resolution of sensors (Li et al., 2019). Figure 6 shows the loading factors associated with PC1 and PC2 for each sensor. At the beginning of storage (Figure 6a), sensors W5W and W1W showed a relatively significant influence on PC1.

Meanwhile, W1W and W2W showed a vital influence on PC2. Other sensors were close to zero point, suggesting minimal contribution. After 60 days of storage, sensors W5S and W1W still showed a relatively significant influence on PC1, and for PC2, W1W was changed significantly, exhibiting a longer distance from zero point than W5S. The sensor W2W also displayed a remarkable influence on PC2. Sensors W5S, W1W, and W2W responding to the odor of chicken meat were not changed at the beginning or end of storage, revealing that the characteristic odor of chicken meat was not shifted markedly after 60 days of storage.

## 4 CONCLUSIONS

In conclusion, we showed that different doses of gamma irradiation significantly decreased TVB and TCC and prolonged the shelf life of spicy chicken meat during storage time. However, irradiation also affected the physicochemical properties of chicken meat. The color and odor of chicken meat were significantly changed in a dose‐dependent manner. In addition, the POV was significantly increased by 6 and 8 kGy of irradiation during the whole storage time, while irradiation had a slight effect on the contents of HAAs. Therefore, we recommended that preservation with gamma irradiation at 4.0 kGy could be an alternative to control the quality of spicy chicken during storage.

## CONFLICT OF INTEREST

We declare that we have no known competing financial interests or personal relationships that could have appeared to influence the work reported in this paper.

## Data Availability

The data that support the findings of this study are available on request from the corresponding author.
